# Identification of the Neural Correlates Underlying Conflict Resolution Performance Using a Rodent Analogue of the Stroop Tests

**DOI:** 10.1016/j.neuroscience.2023.05.024

**Published:** 2023-08-01

**Authors:** S.P. Clinch, M. Busse, J. Griffiths, A.E. Rosser, M.J. Lelos

**Affiliations:** a Brain Repair Group, School of Biosciences, Cardiff University, Cardiff CF10 3AX, UK; b Centre for Clinical Trials Research, School of Medicine, Cardiff University, Cardiff CF24 4HQ, UK

**Keywords:** stroop test, conflict resolution, striatum, cognition, behaviour

## Abstract

The Stroop test is a widely used neuropsychological test measuring attention and conflict resolution, which shows sensitivity across a range of diseases, including Alzheimer’s, Parkinson’s and Huntington’s diseases. A rodent analogue of the Stroop test, the Response-Conflict task (rRCT), allows for systematic investigation of the neural systems underpinning performance in this test. Little is known about the involvement of the basal ganglia in this neural process. The aim of this study was to use the rRCT to determine whether striatal subregions are recruited during conflict resolution processing. To achieve this, rats were exposed to Congruent or Incongruent stimuli in the rRCT and the expression patterns of the immediate early gene *Zif268* were analysed throughout cortical, hippocampal and basal ganglia subregions. The results confirmed the previously reported involvement of prefrontal cortical and hippocampal regions, as well as identifying a specific role for the dysgranular (but not granular) retrosplenial cortex in conflict resolution. Finally, performance accuracy correlated significantly with reduced neural activation in the dorsomedial striatum. Involvement of the basal ganglia in this neural process has not previously been reported. These data demonstrate that the cognitive process of conflict resolution requires not only prefrontal cortical regions, but also recruits the dysgranular retrosplenial cortex and the medial region of the neostriatum. These data have implications for understanding the neuroanatomical changes that underpin impaired Stroop performance in people with neurological disorders.

## Highlights

• We explored the brain regions activated in a rodent version of the Stroop task.
• Our data revealed novel involvement of cortical and striatal regions.
• The dorsomedial striatum, infralimbic and retrosplenial cortices are implicated.
• These data have implications for people with neurological conditions.

## Introduction

The Stroop assessment is a bi-conditional discrimination test which requires a subject to select task relevant information whilst ignoring task irrelevant stimuli (Stroop, 1935, Bullard et al., 2012). This test is commonly used to assess executive functions such as attention and conflict resolution. In the classic Stroop test, subjects are required to either read colour-words, written in their coloured ink (e.g., BLUE in the colour blue), or say the coloured ink of the word which do not correspond (e.g., RED in the colour green). This test has revealed impairments in a wide range of neurological disorders, including Parkinson’s (Djamshidian et al., 2011), Alzheimer’s (Yun, 2011), Huntington’s (HD) (Hunefeldt et al., 2020), glucocerebrosidase gene carriers (Moran, 2021), schizophrenia (Henik and Salo, 2004) and ADHD (Yasumura, 2014, Yasumura, 2019).

The Stroop test is highly sensitive to cognitive decline in HD, even during premanifest stages (Barker, 2013) when striatal atrophy is the most pronounced region of degeneration. This raises the question as to whether the striatum may be involved in conflict resolution processes. Indeed, the demonstration that progressive striatal degeneration in HD correlates with cognitive deficits lends some support to this idea (Sanchez-Pernaute, 2000, Wilkes, 2019). Thus, the aim of this study was to determine whether the striatum is implicated in the conflict resolution processes that critically underpin cognitive function, with specific focus on ventral, medial and lateral striatal subregions. Neuroimaging studies have highlighted several regions of the brain activated during successful performance of the Stroop test, with the majority of focus on the role of the dorsolateral prefrontal cortex (DLPFC) and anterior cingulate cortex (ACg) (Egner and Hirsch, 2005, Harrison, 2005, Ali et al., 2010). Thus, as a secondary goal, we also aim to validate the involvement of regions previously reported to be relevant to this task.

A rodent response conflict task (rRCT) has been developed to recruit the same neural processes as those underlying performance in the human Stroop test (Haddon and Killcross, 2005). This operant test assesses a rodent’s ability to follow task-specific rules by suppressing task-irrelevant stimuli. To achieve this, two audio (A1 or A2) and two visual stimuli (V1 or V2) are presented and are each associated with either a left or right lever. The type of stimulus presented (audio or visual) is associated with one of two contexts (C1 or C2) in the operant boxes. After training, rats are tested using either ‘congruent’ or ‘incongruent’ stimuli. In the congruent scenario, both the auditory and visual stimuli correspond to the same lever response. In the incongruent condition, the audio-visual stimuli presented are associated with different lever responses. The rat is required to disambiguate the audio-visual stimuli by attending to the context it is placed in (i.e., the ‘rule’), thereby suppressing the tendency to respond to the opposing lever.

Previous lesion studies suggest that involvement of the medial prefrontal cortex (mPFC) is required for successful performance in the rRCT. This is understood to be most analogous to primate DLPFC, and can be sub-divided into four regions: the anterior cingulate cortex (ACg), prelimbic cortex (PrL), infralimbic cortex (IL) and precentral cortices. Specifically, rats with PrL, IL and ACg lesions all performed worse in the rRCT either through ablation of goal directed behaviour, lack of inhibitory control or lack of cognitive control (Haddon and Killcross, 2005, Marquis Killcross et al., 2007, Dwyer et al., 2010, Oualian and Gisquet-Verrier, 2010, Haddon and Killcross, 2011). Additional studies revealed that lesions to the retrosplenial cortex (RSC), impaired performance in the incongruent trials of the rRCT (Nelson et al., 2014), whereas no performance differences were seen in rats with hippocampal lesions (Haddon and Killcross, 2007). However, the involvement of subregions of the striatum in this task has yet to be investigated.

In order to identify neural circuits activated by learning or memory recall, molecular mechanisms of neural plasticity can be exploited, such as visualising the rapid upregulation of immediate early genes (IEG) that encode inducible, regulatory transcription factors. One such IEG, *Zif268* (zinc finger protein 268, also known as EGR-1 (early growth response protein 1)), has long been implicated in neural plasticity changes associated with learning and memory (Bozon, 2003, Veyrac et al., 2014, Duclot and Kabbaj, 2017). Therefore, to understand the neural activity patterns associated with cognitive conflict resolution processes, we tested rats on congruent and incongruent versions of the rRCT task and brain tissues were harvested to visualise Zif268 upregulation in cortical, hippocampal and striatal regions.

To achieve this, healthy rats were trained in the rRCT and then were split into two groups and assessed in separate tests of congruent or incongruent stimuli. At 90 min after the test session, rats were culled via transcardial perfusion and brain tissue harvested for immunohistochemical analysis. A third group of naïve rats were included as cage controls; naïve control rats provided baseline measures of Zif268 expression, allowing for task-induced changes in Zif268 to be detected in tested rats. Analysis of Zif268 was used to map patterns of neural activation in rRCT task; use of separate congruent and incongruent test sessions allowed for identification of (1) the specific patterns of neural activation in the simple congruent associative test, and (2) the patterns of neural activation associated with conflict resolution processing in the incongruent test. Prefrontal cortical and hippocampal regions were included in the Zif268 analysis to validate the experimental approach, and striatal subregions were assessed to determine whether these regions were recruited during performance on either the congruent and/or incongruent versions of the cognitive task.

## Experimental procedures

### Subjects

Twenty-four female Lister-hooded rats (Harlan Olac, UK) were included in this experiment, 16 of which underwent operant testing and eight remained as cage controls. Experimental rats for operant testing were housed in groups of four and maintained on ∼12 g rat of chow diet per day to maintain 85–90% of their original ab libitum weights. Cage controls were handled regularly and given food ad libitum for a total of 6 weeks before being perfused. Holding rooms were maintained in a temperature (21^+/-^2°) and humidity (55%^+/-^10%) controlled room at a 14:10-h light/dark cycle. All experiments were conducted in compliance with the UK Animals (Scientific Procedures) Act 1986 under Home Office Licence No. 30/3036 and with the approval of the local Cardiff University Ethics Review Committee.

### Apparatus

Behavioural training took place in a bank of 12 skinner operant boxes (Campden Instruments, Loughborough, UK), controlled by the Cambridge Cognition Control BNC software (Campden instruments, Version 1.23), as described (Dunnett and White, 2006). Each operant box was fixed with two retractable levers fitted either side of a magazine where rats retrieved 45 mg of sucrose pellet rewards dispensed following a correct response. Lights were fitted above each lever, the magazine and a 3 W house light on the ceiling of the operant chamber. Six operant boxes were fitted with a laminated checked context with the smell of cumin, which was mixed with the sawdust in the tray below the floor, and six were fitted with a laminated stripy context with the smell of cinnamon.

### Behavioural training and testing

Behavioural training took place between 09.00 and 17.00, with one training session taking place in the morning (09.00–11.00) in one context and a training session in the other context in the afternoon (14.00–16.00).

**Fig. 1:**
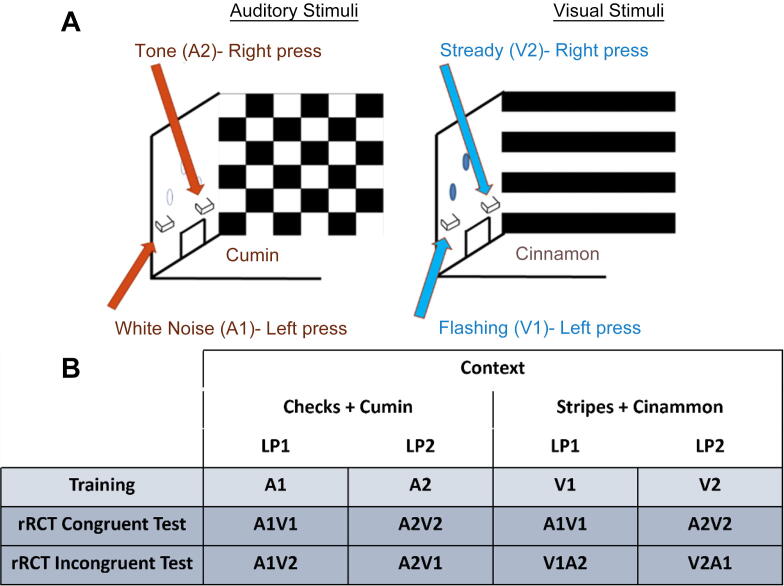
Schematic representation of the rRCT. (**A**) This figure presents a schematic of the operant design for the rRCT during training and testing. It illustrates the two different contexts rats were placed in, which were either associated with two different types of auditory (A1 and A2) or two different types of visual stimuli (V1 and V2). (**B**) ‘Training’ row is an example of stimuli that rats were trained to associate with each lever press in each context. In the test session, the rRCT Congruent Test consisted of pressing the lever associated with the ‘congruent’ audio-visual stimuli. The rRCT Incongruent Test used incongruent audio-visual stimuli (i.e. two stimuli that were previously associated with two different lever press responses during training). In the Incongruent version of the test, rats had to attend to the context (i.e. the ‘rule’) to decipher the correct lever press response. Abbreviations: rRCT = rodent response conflict task; LP1 = Lever press 1 (left lever); LP2 = Lever press 2 (right lever); A1 = auditory stimulus 1 (white noise); A2 = auditory stimulus 2 (tone); V1 = visual stimulus 1 (flashing light); V2 = visual stimulus 2 (steady light).

Task Training: Context/stimuli training: initial operant training and habituation to the operant boxes consisted of one magazine training session and two lever press-training sessions in each context. Operant training on the rRCT was based on the study by Haddon and Killcross (Haddon and Killcross, 2005) with minor modifications. Each training session lasted for 48 min. Stimulus-response associations were fully counterbalanced across all rats. An example of the stimuli presented during a training session is presented in Fig. 1. Context 1 (C1; checked context and cumin) was associated with auditory stimuli (A1 or A2), whereas context 2 (C2; stripy context and cinnamon) was associated with visual stimuli (V1 or V2; Fig. 1(**A**)). Auditory stimuli consisted of either white noise (A1) or tone (A2), and visual stimuli consisted of flashing (V1) or a steady light (V2). Rats were trained to associate each stimulus with either the left or the right lever. Following a correct response, a sucrose pellet reward was dispensed via a lit magazine. Each training session (in either context 1 or 2) consisted of 24 pseudorandom stimulus presentations, 12 of each (either A1 and A2 or V1 and V2). Stimulus presentations lasted for 60 s with 60 s inter-stimulus interval. Rats were trained to learn stimulus–response associations for 23 days until they performed to ≥80% accuracy (Fig. 3(**A**)).

rRCT tests: All rats received eight test sessions (x4 rRCT congruent tests and x4 rRCT incongruent tests), each of which lasted for 25 min and was run in extinction (i.e., in the absence of sucrose rewards). The extinction test is used to probe an ‘extinction burst’; a short increased response in an attempt to achieve the desired reward (Lattal and Lattal, 2012). Rats then received two days of rewarded training (as described below) before the next extinction test day.

For the Congruent test, rats were placed in one of the two contexts and presented with simultaneous audio-visual stimuli followed by the exposure of the left and right levers. In this condition, both stimuli corresponded to the same lever. In the Incongruent test, rats were again exposed to two simultaneous auditory and visual stimuli, but this time each stimulus was associated with opposing levers. To respond correctly, the rat had to utilize the context (‘rule’) and determine which context-stimulus association had been previously learnt. Thus, a ‘correct’ lever press was one that was associated with the stimulus that had previously been paired with the context. To ensure that IEG expression was only induced by performance on the rRCT tests, rats were placed into a quiet, darkened room for a minimum of 90 min before and after each training session.

Final extinction test: Rats were divided into two groups, one of which received the Congruent test as the final test session, while the other group received the Incongruent test as the final session. The two groups were compiled based on accuracy scores from the previous testing sessions. The final test session was run in one context only and the time to cull the rat was determined by the mid-point of the test session. Thus, tissue was harvested 90 min after the central point in the testing session, which coincided with peak Zif268 expression. All rats were placed into a quiet, darkened room before and after the final test session to eliminate the impact of any environmental stimuli on IEG expression.

### Perfusion

Rats were terminally anaesthetized with sodium pentobarbital (Euthatal, Merial, Woking, UK) and sacrificed by transcardial perfusion with 0.01 M phosphate buffered saline (PBS). Each rat received 250 mL of cold 4% paraformaldehyde (PFA) over 5 min. Rats were decapitated and brains removed and stored in 4% PFA for 4 h before transferred into a 25% sucrose solution in PBS at room temperature.

### Immunohistochemistry

Brain tissue was cut coronally into 40 µm thick sections on a freezing microtome and stored at −20 °C in 48 well plates in ethylene–glycol-based cryoprotectant. A 1:12 series of free-floating sections were used for the immunohistochemical assay. Endogenous peroxidases were quenched by incubating sections in 10% methanol, 10% hydrogen peroxide and 80% distilled water. Sections were blocked with 3% normal serum and then incubated in the primary antibody Zif268 (1: 1000; Egr-1 (C-19) Santa Cruz Biotechnology, Santa Cruz, CA, USA) and 1% normal serum in room temperature overnight. The following day sections were incubated with a biotinylated secondary antibody with 1% normal serum for 3 h. The sections were immersed in avidin-biotinylated enzyme complex (Vector Laboratories, Peterborough, UK) with 1% normal serum for 2 h and stained with 3,3′-Diaminobenzidine (DAB) until light brown. Sections were mounted onto gelatinized glass slides and left to air dry overnight. The sections were dehydrated in ascending concentration of 70%, 95% and 100% of alcohols and then immersed in xylene. Slides were coverslipped using di-n-butyl phthalate in xylene (DPX) mounting medium and air-dried.

**Fig. 2:**
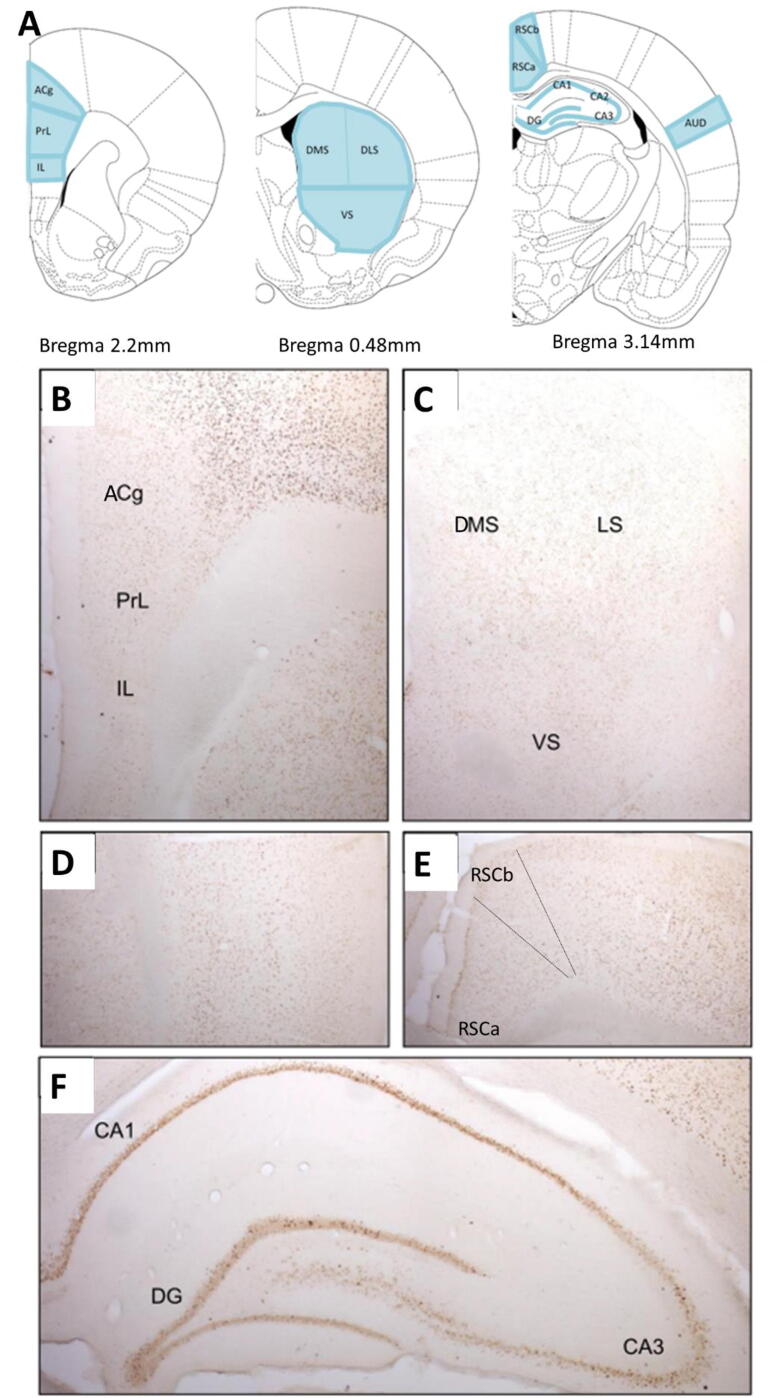
(**A**) Coronal sections depicting brain regions of interest for immunohistochemical analysis based on Paxinos and Watson Rat Brain Atlas. Representative images of Zif268 staining in subregions of the prefrontal cortex (**B**), striatum (**C**), auditory cortex (**D**), retrosplenial cortex (**E**) and hippocampus (**F**). ACg = anterior cingulate cortex; AUD = auditory cortex; DG = dentate gyrus; DLS = dorsolateral striatum; DMS = dorsomedial striatum; IL = infralimbic cortex; PrL = prelimbic cortex; RSC = retrosplenial cortex; VS = ventral striatum.

Sections were visualized using a brightfield Leica DMRB microscope with x5 objective and captured using an Olympus DP70 camera on Leica Application Suite Imaging software. Some images were captured and stitched together using Windows Live Photo Gallery. Representative images are presented in Fig. 2(**B-F**).

**Fig. 3:**
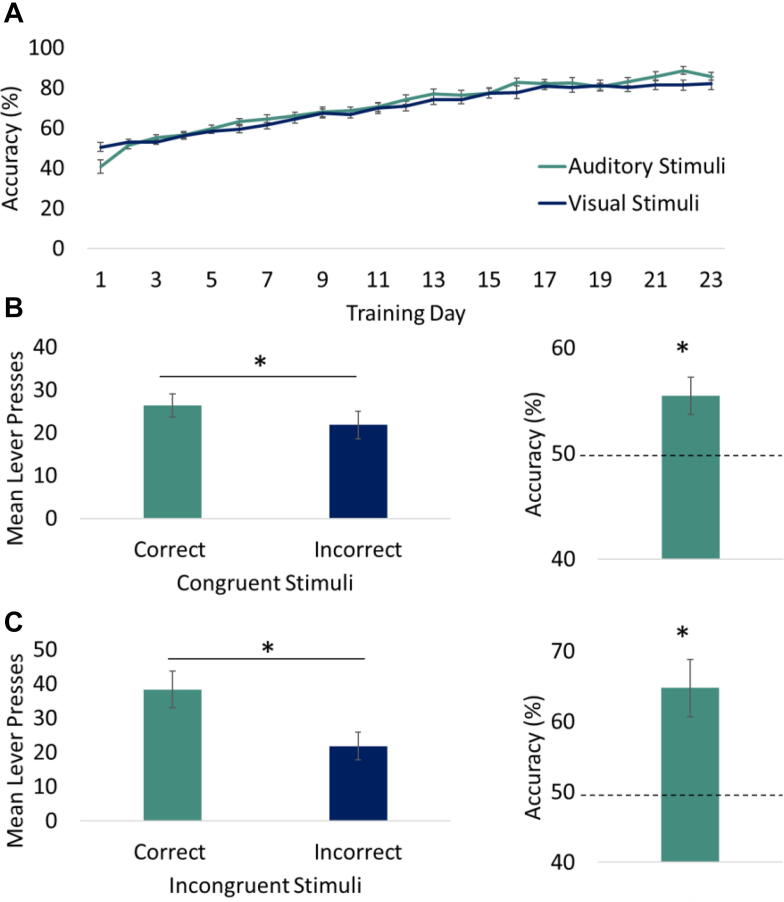
Behavioural performance in the rRCT. (**A**) Rats were trained on the rRCT for 23 days and all rats achieved > 80% accuracy for both auditory and visual stimuli. (**B**) In the Congruent extinction test, significantly more correct than incorrect responses were made and accuracy performance was greater than chance. (**C**) In the Incongruent extinction test, significantly more correct than incorrect responses were made and accuracy performance was greater than chance.

### Image J analysis

All images were analysed blind to the experimental group allocation. IEG expression was quantified using Image J software (Version 1.51, National Institutes of Health, USA). To quantify the number of immunoreactive nuclei, an individual threshold was set for each region of interest and remained consistent for all rats. A watershed was applied and the number of immunoreactive positive nuclei were counted in set area and averaged across both hemispheres. The number of immunoreactive positive (IR) nuclei per 10 µm^2^ were calculated using the equation:

$$100*\frac{\left(Average\;number\;of\;IR\;positive\;nuclei\right)}{Average\;area}=Average\;IR\;nuclei/10\;{\mu m}^{2}$$

### Regions of interest

Cytoarchitectonic subfields of the prefrontal cortex (prelimbic cortex (PrL), infralimbic cortex (IL) and anterior cingulate cortex (ACg)), striatum (dorsolateral (DLS), dorsomedial (DMS) and ventral (VS)), hippocampus (CA1, CA2, CA3 and dentate gyrus (DG)), retrosplenial cortex (granular (RSCa) and dysgranular (RSCb)) and the auditory cortex were identified in all sections using nomenclature based on Paxinos and Watson’s Rat Brain Atlas (Paxinos and Watson, 1986) (Fig. 2(**A**)). Immunoreactive cells were also counted in the auditory cortex, which was used as an internal control to validate the IEG staining specifically between rRCT groups (rRCT incongruent and rRCT control) and naïve controls. It was anticipated that higher Zif268 immunoreactivity would be evident in the auditory cortex of the rRCT groups, relative to cage Controls, due to the exposure to auditory stimuli in the operant boxes.

The neuroanatomical coordinates used for image analysis were between 4.7 mm to 2.70 mm from bregma for all prefrontal cortical regions; 1.60 mm to −1.40 mm from bregma for all striatal regions; −2.30 mm to −4.30 mm from bregma for all hippocampal subregions; −1.60 mm to −4.52 mm from bregma for the retrosplenial cortex; −3.14 mm to −4.42 mm from bregma for the auditory cortex.

### Statistical analysis

One rat was perfused before the end of the experiment due to illness. An additional two rats were removed from experiment due to an inability to learn the behavioural task (one rat) and inconsistent immunostaining (one rat). All remaining rats in the naive control (n = 8), rRCT control (n = 7) and rRCT incongruent group (n = 6) completed the experiment and were included in the analysis. SPSS version 25 (IBM Corporation, USA) was used to analyse the data. For all behavioural training data, performance during the first 30 s of stimuli presentation was used, as published previously (Haddon and Killcross, 2006). The final extinction test data were based the first 12 min of the test, which was chosen to capture vigorous responding and was coordinated with peak Zif268 activation (Chaudhuri et al., 1995, Kaminska et al., 1996). Training data were analysed by ANOVA with Stimulus (Auditory, Visual) and Day (1–23) as factors. Post-hoc tests were conducted using Sidak’s correction for multiple comparisons. Performance in the final rRCT Congruent and Incongruent tests was analysed using independent t-tests. For the IEG counts, the mean number of immunoreactive positive nuclei per µm^2^ were analysed by one-way ANOVA with the factor of Group (Cage Control, rRCT Congruent or rRCT Incongruent). A Pearson’s correlation was used to assess the relationship between IEG expression and performance accuracy in the final task. All statistical analyses used *p* < 0.05 to reject the null hypothesis.

## Results

### Behavioural results

**Fig. 4:**
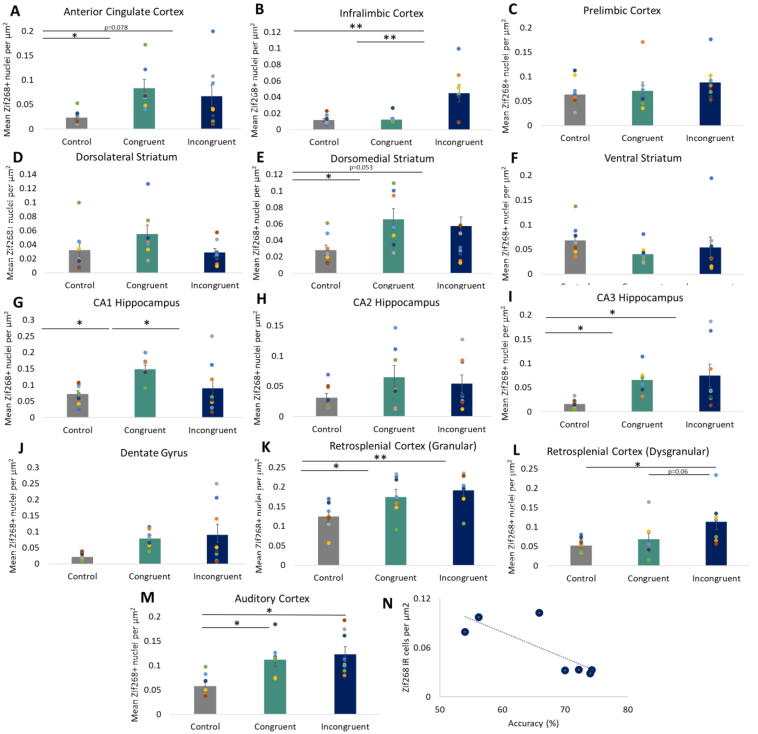
The mean number of nuclei immunoreactive for Zif268 in subregions of the prefrontal cortex (**A-C**), striatum (**D-F**), hippocampus (**G-J**), retrosplenial cortex (**K, L**) and auditory cortex (**M**). Correlation between accuracy on the incongruent task and Zif268 immunoreactivity in the DMS (**N**). Error bars show ± standard error of the mean. *p < 0.05, **p < 0.01.

rRCT Training: All rats were trained to a high level of accuracy, successfully responding to the correct lever following stimulus presentation with than 80% accuracy for both auditory and visual stimuli (Fig. 4(**A**)).

Congruent and Incongruent Tests: Analysis of the mean performance on the Congruent and Incongruent probe tests revealed significantly more correct than incorrect lever responses during the stimulus presentations (both ps < 0.05, respectively; Fig. 3(**B,C**)). Moreover, comparison of the mean percentage accuracy to 50% (chance) confirmed accurate performance for both tests (ps < 0.05).

### Zif268 IEG results

Representative images of Zif268 staining are depicted in Fig. 2(**B-F**).

**Prefrontal cortex**. As anticipated, group differences in Zif268 expression were identified in subregions of the mPFC. In the ACg, there was a significant upregulation of Zif268 between naive controls and rRCT rats [Group: F_2,22_ = 3.49, *p* = 0.05; naive controls vs rRCT Congruent *p* < 0.02; naïve controls vs rRCT Incongruent *p* = 0.078; Fig. 4(**A**)]. In the IL cortex, there was a notable upregulation of Zif268 in the rRCT Incongruent group compared to both naive controls and rRCT Congruent rats [Group: F_2,22_ = 8.51, *p* = 0.002; rRCT Incongruent vs both naive controls and rRCT Congruent rats, both ps < 0.01; Fig. 4(**B**)]. No difference in IEG expression was evident between groups for the PrL [Group: F_2,22_ = 0.09, n.s.; Fig. 4(**C**)].

**Striatum**. More immunoreactive nuclei were identified in the DMS of rats that underwent rRCT than naïve controls [F_2,22_ = 3.68, *p* < 0.05; naïve controls versus rRCT Congruent *p* < 0.05; naïve controls versus rRCT Incongruent *p* = 0.053; Fig. 4(**E**)]. Zif268 expression did not differ in the DLS and VS between any of the groups [Group: F_2,22_ = 2.01 and 0.85, respectively; Fig. 4(**D,F**)].

**Hippocampus**. rRCT performance induced zif268 expression in the CA3 generally [Group: F_2,22_ = 4.43, *p* < 0.05; naïve vs both rRCT Congruent and Incongruent groups, ps < 0.05; Fig. 4(**I**)]. Zif268 expression was specifically increased in the CA1 region for rRCT Congruent only [Group: F_2,22_ = 4.02, *p* < 0.05; rRCT Congruent vs naïve and rRCT Incongruent groups, ps < 0.05; Fig. 4(**G**)]. No differences were observed in the CA2 or DG regions [Group: F_2,22_ = 1.46 and 3.20, *p* = n.s. and 0.062, respectively; Fig. 4(**H,J**)].

**Retrosplenial cortex**. For the dysgranular RSCb, Zif268 expression was significantly upregulated in the rRCT Incongruent group [Group: F_2,22_ = 4.17, *p* < 0.05; rRCT Incongruent vs naive controls *p* < 0.05; rRCT Incongruent vs rRCT Congruent *p* = 0.06; Fig. 4(**L**)]. More IR cells were evident in the granular RSCa in both rRCT groups [Group: RSCa, F_2,22_ = 4.97, *p* < 0.05; naïve Controls vs both rRCT groups, ps < 0.05; Fig. 4(**K**)].

**Auditory cortex**. Zif268 expression was upregulated in both rRCT groups relative to naïve Controls [Group: F_2,22_ = 7.90, *p* < 0.01; naïve Controls vs both rRCT groups, ps < 0.01; Fig. 4(**M**)].

**Correlations:** Zif268 immunoreactivity in the DMS correlated negatively with performance on the incongruent version of the rRCT [*r*^2^ = −0.794, *p* < 0.05; Fig. 4(**N**)]. No significant correlations were evident between other brains regions and accuracy in the incongruent task [DLS: *r*^2^ = −0.031; VS: *r*^2^ = 0.548; ACg: *r*^2^ = −0.677; PRL: *r*^2^ = −0.643; IL: *r*^2^ = −0.187; CA1: *r*^2^ = 0.081; CA2: *r*^2^ = −0.048; CA3: *r*^2^ = −0.190; DG: *r*^2^ = −0.044; RSCa: *r*^2^ = −0.166; RSCb: *r*^2^ = −0.418; Aud: *r*^2^ = 0.397; all ps = n.s.].

Performance in the rRCT congruent test correlated negatively with both DMS and ACg Zif268 expression [*r*^2^ = −0.863 and −0.783, respectively, ps < 0.05]. No significant correlations were evident between other brains regions and accuracy in the congruent task [DLS: *r*^2^ = −0.613; VS: *r*^2^ = −0.118; PRL: *r*^2^ = −0.332; IL: *r*^2^ = 0.225; CA1: *r*^2^ = 0.387; CA2: *r*^2^ = 0.078; CA3: *r*^2^ = −0.108; DG: *r*^2^ = −0.133; RSCa: *r*^2^ = 0.338; RSCb: *r*^2^ = 0.444; Aud: *r*^2^ = −0.102; all ps = n.s.].

## Discussion

Thus, the aim of the study was to identify the neural regions recruited during response conflict processing, using the operant rRCT and IEG imaging techniques. Of all the cortical, striatal and hippocampal regions probed in this study, we demonstrate specific involvement of the IL cortex in conflict resolution performance, as well as a relationship between response accuracy and DMS striatal activity. There is also a trend for higher IEG activity in the dysgranular region of the RSC (*p* = 0.06). The Stroop test has been used to detect deficits in executive function, with known sensitivity to attentional impairments and impaired cognitive processing, specifically conflict resolution in the presence of competing stimuli. Impairments in Stroop performance have been reported in patients with a range of neurological conditions, including Parkinson’s, Huntington’s, Alzheimer’s, stroke, schizophrenia and ADHD. Several studies have established the sensitivity of this test to detecting mPFC dysfunction in both clinical populations and preclinical rodent experiments (Egner and Hirsch, 2005, Haddon and Killcross, 2005, Harrison, 2005, Marquis Killcross et al., 2007). Basal ganglia dysfunction also manifests early and as a predominant feature in many of the same clinical populations, including Parkinson’s and Huntington’s diseases. This raised the question as to whether the basal ganglia may be recruited during the Stroop test performance and, specifically, whether this test may detect striatal dysfunction.

During the final extinction test, rats in the rRCT Incongruent group were required to disambiguate audio-visual stimuli based on the context in which they were placed. Rats in the rRCT Incongruent group achieved significantly more correct than incorrect responses, supporting previous studies that rats can successfully identify and respond to conflicting information (Haddon and Killcross, 2005, Haddon and Killcross, 2006, Marquis Killcross et al., 2007, Haddon and Killcross, 2011). One difference between the current study and previously published studies is the higher accuracy scores obtained in the Incongruent version of the task, whereas greater accuracy is typically reported on the Congruent test. As noted in the methods, the rats were divided into groups for the final test based on their previous performance. Thus, while rats were included in the Incongruent group that had achieved decent performance on the Incongruent tests, it was the case that several (∼4) rats performed particularly well in the final task, making the mean for this group higher than the Congruent test. Although there was some variability in performance during the final test session, these differences could not be identified during training. This may suggest that variability in final test performance was not determined by superior learned discrimination in individual animals, but since the training data were also very consistent between animals (i.e., lacked variability), this dataset allowed little opportunity to discriminate between animals that formed stronger versus weaker associations during training. Thus, the rodents that performed in the Incongruent test were robustly able to resolve the biconditional conflict, which should provide confidence that analysis of these brains is appropriate for identifying correlations with performance and for identifying brain regions involved in conflict resolution. Indeed, this is supported by our data confirming the anticipated involvement of the infralimbic cortex and retrosplenial cortex.

Changes in the neuronal IEG Zif268 has previously been used to identify neural activity and recruitment of specific brain regions during learning or memory retrieval (Martinez et al., 2002, Rygh, 2006). The IEG imaging identified involvement of mPFC, auditory cortex and hippocampal regions, each of which was included in order to validate the experimental approach. Indeed, significant changes in Zif268 activity in the prefrontal cortex (IL and ACg), hippocampus (CA1, CA3), retrosplenial cortex (RSCa and RSCb) and the auditory cortex were identified between Cage Controls and animals performing either version of the rRCT task. However, the Incongruent rRCT task specifically recruited the IL cortex and showed a trend for the dysgranular region of the RSC (*p* = 0.06). Zif268 expression in the dorsomedial striatum also correlated with performance accuracy in both the rRCT Incongruent groups.

In this study, changes in IEG expression in subregions of the PFC in rRCT groups were observed. There was a significant upregulation of Zif268 positive nuclei in the IL cortex in the rRCT Incongruent group relative to Cage Controls, but no difference between rRCT controls and Cage Controls. This suggests that the IL was recruited during conflict resolution processing in the rRCT Incongruent task. This contrasts with findings from a previous finding that used the rRCT and revealed that temporary inactivation of the IL did not affect the accuracy of performance in the incongruent test (Marquis Killcross et al., 2007). However, other studies had revealed different roles for the IL cortex including behavioural flexibility (Oualian and Gisquet-Verrier, 2010) and extinction learning (Barker et al., 2014), whilst others have shown it is fundamental for fixed behaviour such as habits (Marquis Killcross et al., 2007). Thus, further studies are needed to disambiguate the role of the IL in conflict resolution performance or extinction learning aspects of this task.

The results in this study also showed an upregulation of Zif268 in the ACg in both rRCT groups relative to Cage Controls, suggesting that these regions may have been recruited more generally as a response to exposure to the stimuli and/or motor responses, rather than conflict resolution per se. This finding is somewhat surprising as numerous studies suggest that the ACg is involved when evaluating conflict scenarios (Sachuriga, 2021). However, the ACg has also been implicated in the evaluation of action-outcomes and goal-directed behaviour in response to locomotion (Botvinick et al., 2004, Sachuriga, 2021), which may explain the pattern of Zif268 upregulation in this region.

Counts recorded from the hippocampus revealed significant changes in Zif268 activation in the CA1 and CA3 subregions between Cage Controls and the rRCT groups. This supports previous findings that suggest the CA1 and CA3 subregions of the hippocampus are involved in associative learning, specifically pairing a stimulus to a context (Izuma et al., 2010), but not necessarily for conflict resolution (Haddon and Killcross, 2007).

Changes in Zif268 expression in the RSC were identified between Cage Controls and both rRCT groups. Interestingly, Zif268 upregulation was observed in the rRCT Incongruent group relative to Cage Controls, but no difference in the rRCT control group. This supports a previous finding that suggest the RSC is involved in conflict resolution (Nelson et al., 2014). Nelson et al. report successful learning of context-dependent rules after damage to the RSC, but difficulty in selecting task relevant stimuli (Nelson et al., 2014). However, the RSCa and RSCb subregions were not assessed independently in this study. The RSCb is more extensively connected with cortical and subcortical regions than RSCa (Hindley et al., 2014), which may be relevant to processes involved in conflict resolution.

In this study, we asked whether the striatum is necessary for conflict resolution. Striatal degeneration occurs early in Huntington’s disease and a previous study reported that striatal degeneration correlated with performance accuracy in the incongruent Stroop test (Sanchez-Pernaute, 2000, Ali et al., 2010). As striatal involvement has not yet been investigated in the rRCT, analysing the potential involvement of this region during a conflicting scenario was of particular interest. The results indicate that no changes were evident in general Zif268 expression in the DLS or VS subregions of the striatum, but the DMS was recruited during performance in both versions of the task. Correlation analysis, furthermore, suggests that increased Zif268 expression is decreased in accordance with more accurate performance on the Congruent and Incongruent tests. The DMS is analogous to the human caudate and anterior putamen, and has previously been implicated in decision making and in particular goal directed behaviour (Yin et al., 2005, MacDonald, 2014).

In Huntington’s disease, as the disease progresses, the greatest changes appear to be in the less cognitively demanding tests, specifically in the time taken to recite colour names and words read, i.e., the congruent test (Snowden et al., 2001, Ho, 2003). An explanation for this could be that information processing speed and recitation of automatic over-learned sequences deteriorates as the disease progresses, which is thought to involve a striatal contribution (Cansler, 2023) Therefore, the data from this study shows that the striatum may be globally important for performance in the rRCT task, which may explain why altered Zif268 expression in the DMS was observed in both the rRCT Congruent and Incongruent groups, albeit that lower expression was actually associated with greater accuracy. However, further validation of the roles of the IL, DMS and RSCb in response confliction are required via lesion or inactivation studies.

It is worth noting that female rats are used in the present study, which is in contrast to the previous studies that have used this task (Haddon and Killcross, 2005, Haddon and Killcross, 2006, Haddon and Killcross, 2007, Marquis Killcross et al., 2007, Haddon and Killcross, 2011, Nelson et al., 2014). Recently, there has been considerable discussion of the importance of using box sexes in research, to ensure that any sex differences in neurochemistry or behaviour are recognised and that biomedical studies consider any potential differential impacts of therapeutics, in terms of dosing or hormone interactions, for example. The primary concern regarding the use of female rodents has often been that oestrus cycles affect performance on behavioural tasks. However, there have been several meta-analyses that report that the use of female rodents in behavioural studies does not introduce any greater variability due to oestrous cycling (Becker et al., 2016, Beery, 2018, Kaluve et al., 2022). Indeed, our data are broadly similar to those reported in studies using males rats (Haddon and Killcross, 2005, Haddon and Killcross, 2006, Marquis Killcross et al., 2007, Haddon and Killcross, 2011, Nelson et al., 2014), which suggests that this biconditional task can be used effectively with either sex. Since we have not directly compared performance between the two sexes, however, it remains to be explore whether this factor has any impact on performance.

Finally, it is worth noting the medial prefrontal cortex is highly integrated with the olfactory system (Cansler, 2023). Thus, it is possible that IEG activation in the medial prefrontal cortex could be induced through attending to olfactory guidance cues. The roles of mPFC subregions have been explored previously (Haddon and Killcross, 2005, Haddon and Killcross, 2006, Marquis Killcross et al., 2007, Haddon and Killcross, 2011) and the IL cortex in particular is implicated in the performance on this task. Thus, despite correspondence between the lesion/inactivation studies and the IEG data presented here, there remains the possibility that IEG activity within the mPFC regions is mediated by exposure to olfactory cues. This could be mitigated in future studies by using alternative experimental designs that do not use olfactory cues (e.g. (Haddon and Killcross, 2005, Marquis Killcross et al., 2007, Haddon and Killcross, 2011, Haddon and Killcross, 2006) rather than the version of the task that incorporates these extra cues (Nelson et al., 2014).

To conclude, this study revealed novel activation of the IL and a trend for the RSCb regions in conflict resolution and correlation between DMS activation and response accuracy on the incongruent task. These data further our understanding of the neurobiological underpinnings of response conflict performance that is inherent to the Stroop test and, although further validation is required, may shed some light on the neural systems that are dysfunctional in early Huntington’s disease and other neurological conditions. Moreover, these data may inform the way that rehabilitation or training interventions are undertaken in neurological disorders, such as Huntington’s disease.

## Acknowledgements

The authors report no conflicts of interest. This study was supported by a Parkinson’s UK Senior Fellowship (F1502) to M Lelos; by Repair-HD, a European Union's Seventh Framework Programme for research, technological development and demonstration [under grant agreement n°602245 (http://www.repair-hd-eu)] and by a MRC Programme Grant MR/T033428/1 to MJL and AER.

## Data Availability

Data are freely available upon resonable request.
